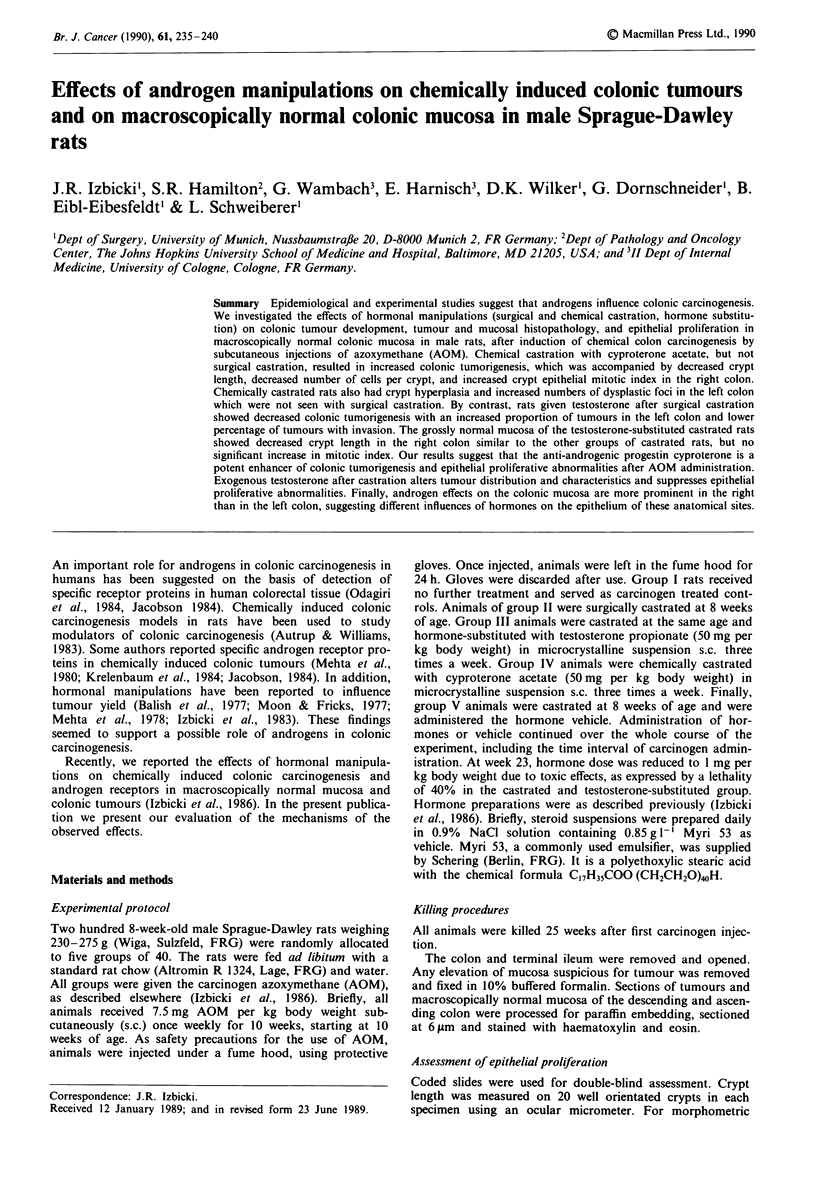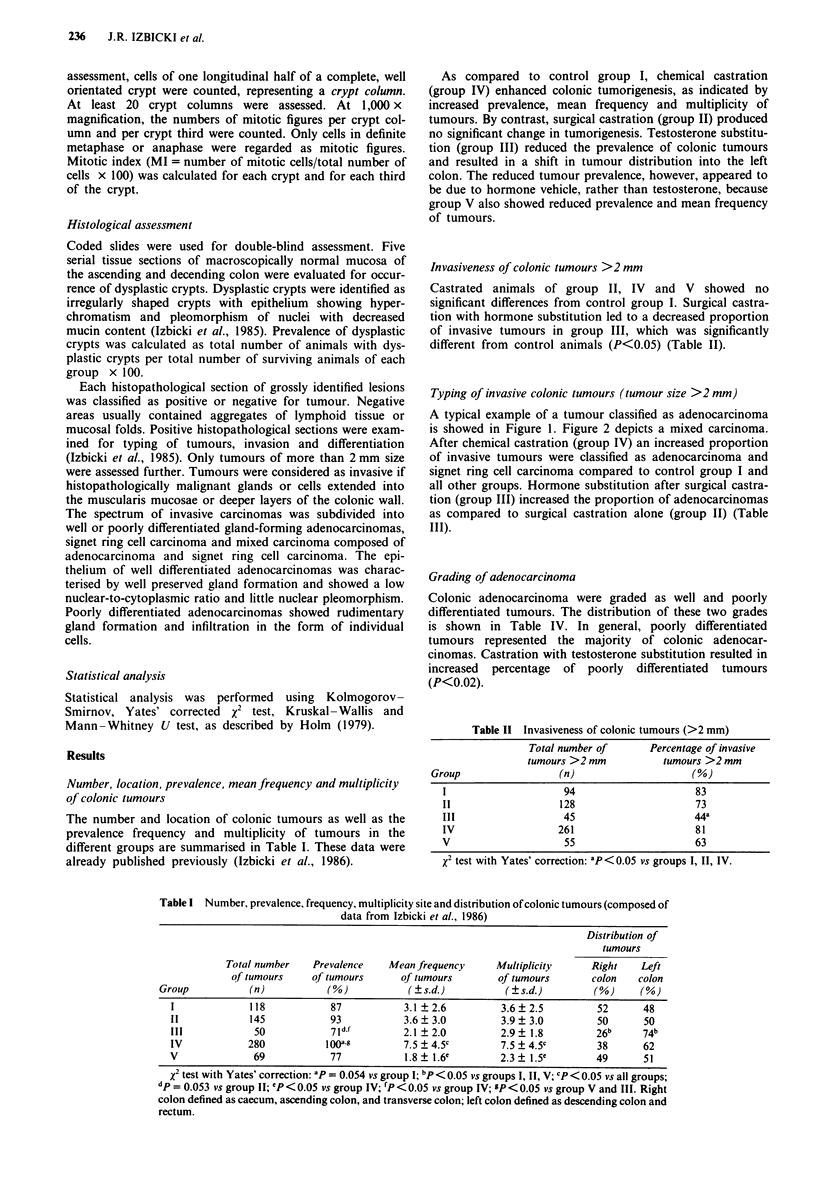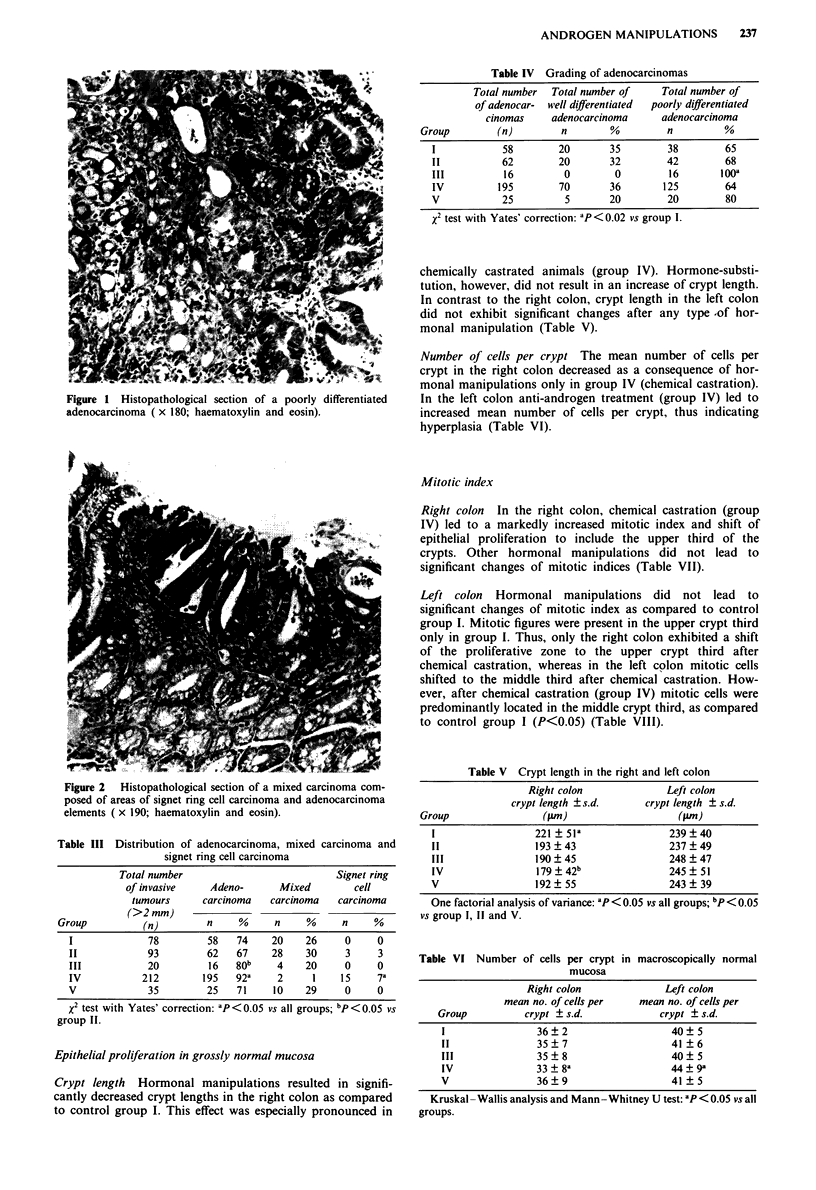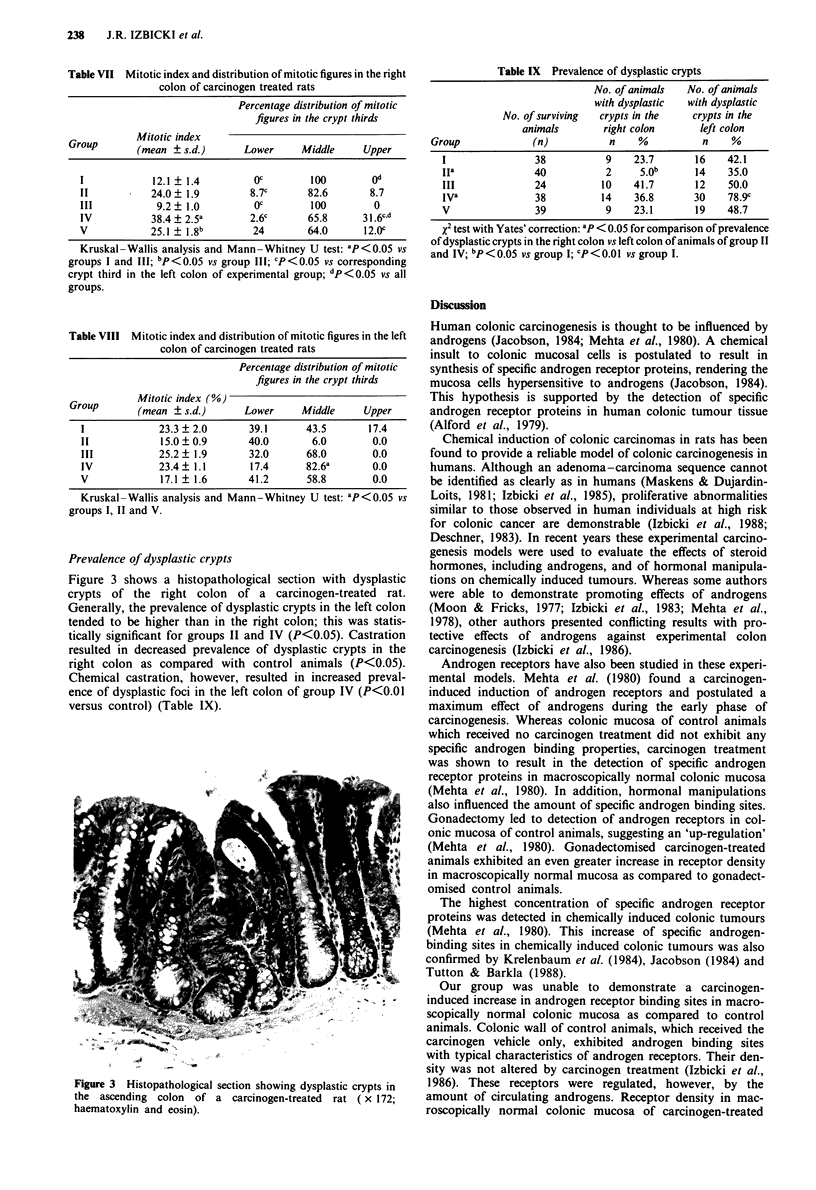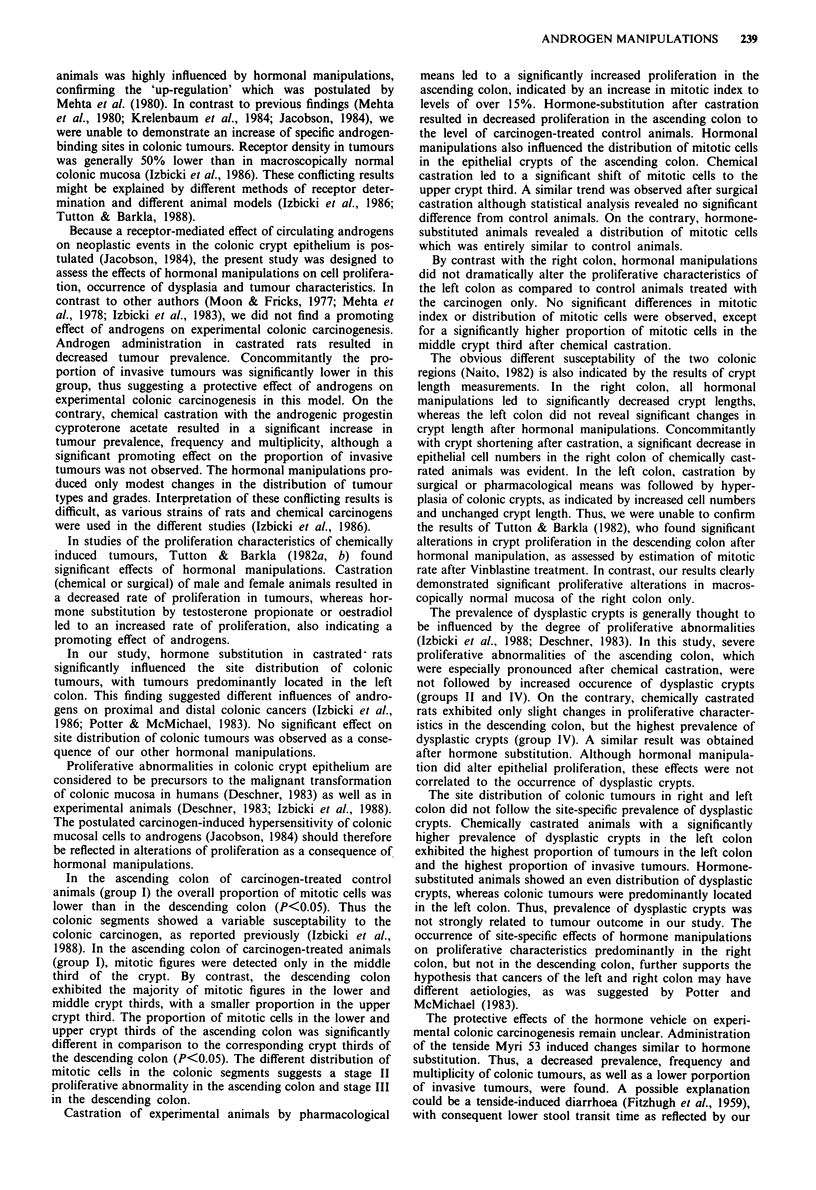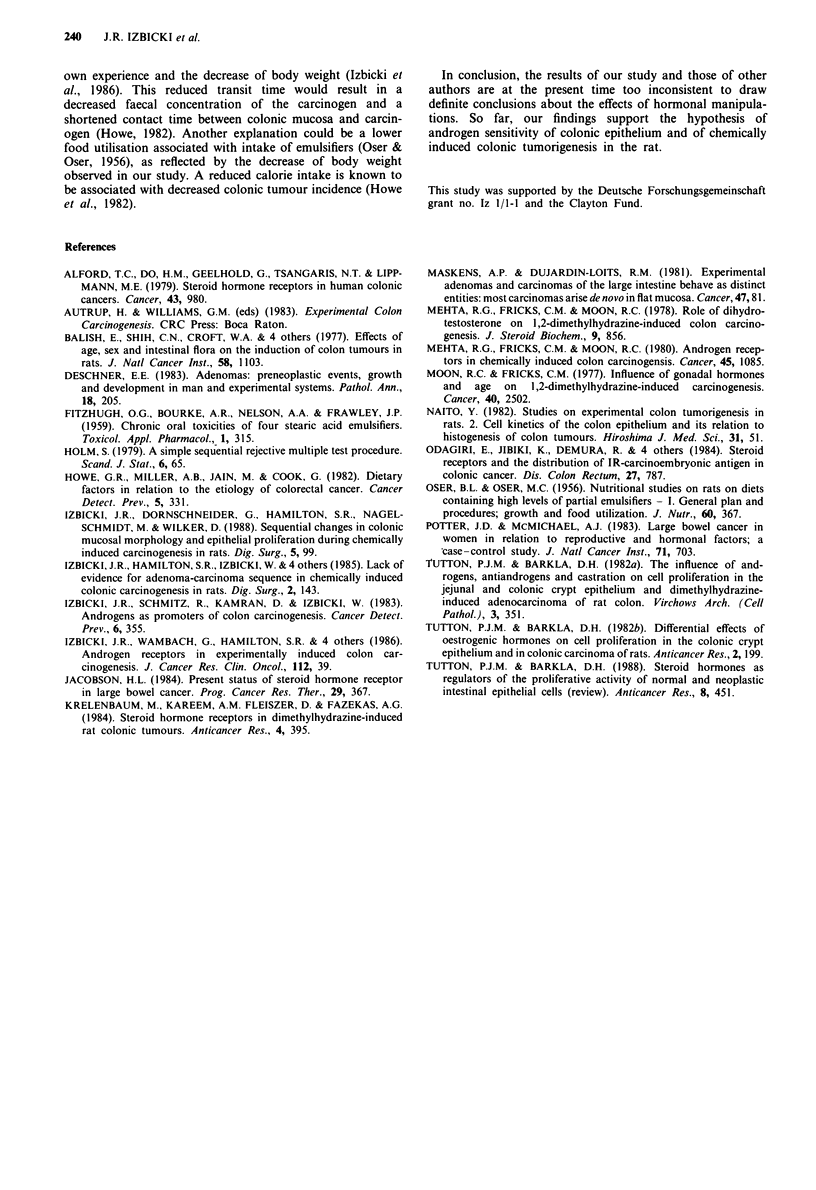# Effects of androgen manipulations on chemically induced colonic tumours and on macroscopically normal colonic mucosa in male Sprague-Dawley rats.

**DOI:** 10.1038/bjc.1990.44

**Published:** 1990-02

**Authors:** J. R. Izbicki, S. R. Hamilton, G. Wambach, E. Harnisch, D. K. Wilker, G. Dornschneider, B. Eibl-Eibesfeldt, L. Schweiberer

**Affiliations:** Dept of Surgery, University of Munich, FR Germany.

## Abstract

**Images:**


*Br. J. Cancer* (1990), **61**, 235–240

© Macmillan Press Ltd., 1990

### Effects of androgen manipulations on chemically induced colonic tumours and on macroscopically normal colonic mucosa in male Sprague-Dawley rats

J.R. Izbicki[1], S.R. Hamilton[2], G. Wambach[3], E. Harnisch[3], D.K. Wilker[1], G. Dornschneider[1], B. Eibl-Eibesfeldt[1] & L. Schweiberer[1]

[1]*Dept of Surgery, University of Munich, Nussbaumstraße 20, D-8000 Munich 2, FR Germany;* [2]*Dept of Pathology and Oncology Center, The Johns Hopkins University School of Medicine and Hospital, Baltimore, MD 21205, USA; and* [3]*II Dept of Internal Medicine, University of Cologne, Cologne, FR Germany.*

**Summary** Epidemiological and experimental studies suggest that androgens influence colonic carcinogenesis. We investigated the effects of hormonal manipulations (surgical and chemical castration, hormone substitution) on colonic tumour development, tumour and mucosal histopathology, and epithelial proliferation in macroscopically normal colonic mucosa in male rats, after induction of chemical colon carcinogenesis by subcutaneous injections of azoxymethane (AOM). Chemical castration with cyproterone acetate, but not surgical castration, resulted in increased colonic tumorigenesis, which was accompanied by decreased crypt length, decreased number of cells per crypt, and increased crypt epithelial mitotic index in the right colon. Chemically castrated rats also had crypt hyperplasia and increased numbers of dysplastic foci in the left colon which were not seen with surgical castration. By contrast, rats given testosterone after surgical castration showed decreased colonic tumorigenesis with an increased proportion of tumours in the left colon and lower percentage of tumours with invasion. The grossly normal mucosa of the testosterone-substituted castrated rats showed decreased crypt length in the right colon similar to the other groups of castrated rats, but no significant increase in mitotic index. Our results suggest that the anti-androgenic progestin cyproterone is a potent enhancer of colonic tumorigenesis and epithelial proliferative abnormalities after AOM administration. Exogenous testosterone after castration alters tumour distribution and characteristics and suppresses epithelial proliferative abnormalities. Finally, androgen effects on the colonic mucosa are more prominent in the right than in the left colon, suggesting different influences of hormones on the epithelium of these anatomical sites.

An important role for androgens in colonic carcinogenesis in humans has been suggested on the basis of detection of specific receptor proteins in human colorectal tissue (Odagiri *et al.*, 1984, Jacobson 1984). Chemically induced colonic carcinogenesis models in rats have been used to study modulators of colonic carcinogenesis (Autrup & Williams, 1983). Some authors reported specific androgen receptor proteins in chemically induced colonic tumours (Mehta *et al.*, 1980; Krelenbaum *et al.*, 1984; Jacobson, 1984). In addition, hormonal manipulations have been reported to influence tumour yield (Balish *et al.*, 1977; Moon & Fricks, 1977; Mehta *et al.*, 1978; Izbicki *et al.*, 1983). These findings seemed to support a possible role of androgens in colonic carcinogenesis.

Recently, we reported the effects of hormonal manipulations on chemically induced colonic carcinogenesis and androgen receptors in macroscopically normal mucosa and colonic tumours (Izbicki *et al.*, 1986). In the present publication we present our evaluation of the mechanisms of the observed effects.

### Materials and methods

### *Experimental protocol*

Two hundred 8-week-old male Sprague-Dawley rats weighing 230–275 g (Wiga, Sulzfeld, FRG) were randomly allocated to five groups of 40. The rats were fed *ad libitum* with a standard rat chow (Altromin R 1324, Lage, FRG) and water. All groups were given the carcinogen azoxymethane (AOM), as described elsewhere (Izbicki *et al.*, 1986). Briefly, all animals received 7.5 mg AOM per kg body weight subcutaneously (s.c.) once weekly for 10 weeks, starting at 10 weeks of age. As safety precautions for the use of AOM, animals were injected under a fume hood, using protective gloves. Once injected, animals were left in the fume hood for 24 h. Gloves were discarded after use. Group I rats received no further treatment and served as carcinogen treated controls. Animals of group II were surgically castrated at 8 weeks of age. Group III animals were castrated at the same age and hormone-substituted with testosterone propionate (50 mg per kg body weight) in microcrystalline suspension s.c. three times a week. Group IV animals were chemically castrated with cyproterone acetate (50 mg per kg body weight) in microcrystalline suspension s.c. three times a week. Finally, group V animals were castrated at 8 weeks of age and were administered the hormone vehicle. Administration of hormones or vehicle continued over the whole course of the experiment, including the time interval of carcinogen administration. At week 23, hormone dose was reduced to 1 mg per kg body weight due to toxic effects, as expressed by a lethality of 40% in the castrated and testosterone-substituted group. Hormone preparations were as described previously (Izbicki *et al.*, 1986). Briefly, steroid suspensions were prepared daily in 0.9% NaCl solution containing 0.85 g $l^{-1}$ Myri 53 as vehicle. Myri 53, a commonly used emulsifier, was supplied by Schering (Berlin, FRG). It is a polyethoxylic stearic acid with the chemical formula $C_{17}H_{35}COO\,(CH_2CH_2O)_{40}H$.

### *Killing procedures*

All animals were killed 25 weeks after first carcinogen injection.

The colon and terminal ileum were removed and opened. Any elevation of mucosa suspicious for tumour was removed and fixed in 10% buffered formalin. Sections of tumours and macroscopically normal mucosa of the descending and ascending colon were processed for paraffin embedding, sectioned at 6 μm and stained with haematoxylin and eosin.

### *Assessment of epithelial proliferation*

Coded slides were used for double-blind assessment. Crypt length was measured on 20 well orientated crypts in each specimen using an ocular micrometer. For morphometric

Correspondence: J.R. Izbicki.
Received 12 January 1989; and in revised form 23 June 1989.

assessment, cells of one longitudinal half of a complete, well orientated crypt were counted, representing a *crypt column*. At least 20 crypt columns were assessed. At 1,000 × magnification, the numbers of mitotic figures per crypt column and per crypt third were counted. Only cells in definite metaphase or anaphase were regarded as mitotic figures. Mitotic index (MI = number of mitotic cells/total number of cells × 100) was calculated for each crypt and for each third of the crypt.

*Histological assessment*

Coded slides were used for double-blind assessment. Five serial tissue sections of macroscopically normal mucosa of the ascending and decending colon were evaluated for occurrence of dysplastic crypts. Dysplastic crypts were identified as irregularly shaped crypts with epithelium showing hyperchromatism and pleomorphism of nuclei with decreased mucin content (Izbicki *et al.*, 1985). Prevalence of dysplastic crypts was calculated as total number of animals with dysplastic crypts per total number of surviving animals of each group × 100.

Each histopathological section of grossly identified lesions was classified as positive or negative for tumour. Negative areas usually contained aggregates of lymphoid tissue or mucosal folds. Positive histopathological sections were examined for typing of tumours, invasion and differentiation (Izbicki *et al.*, 1985). Only tumours of more than 2 mm size were assessed further. Tumours were considered as invasive if histopathologically malignant glands or cells extended into the muscularis mucosae or deeper layers of the colonic wall. The spectrum of invasive carcinomas was subdivided into well or poorly differentiated gland-forming adenocarcinomas, signet ring cell carcinoma and mixed carcinoma composed of adenocarcinoma and signet ring cell carcinoma. The epithelium of well differentiated adenocarcinomas was characterised by well preserved gland formation and showed a low nuclear-to-cytoplasmic ratio and little nuclear pleomorphism. Poorly differentiated adenocarcinomas showed rudimentary gland formation and infiltration in the form of individual cells.

*Statistical analysis*

Statistical analysis was performed using Kolmogorov–Smirnov, Yates' corrected $\chi^2$ test, Kruskal–Wallis and Mann–Whitney *U* test, as described by Holm (1979).

### Results

*Number, location, prevalence, mean frequency and multiplicity of colonic tumours*

The number and location of colonic tumours as well as the prevalence frequency and multiplicity of tumours in the different groups are summarised in Table I. These data were already published previously (Izbicki *et al.*, 1986).

As compared to control group I, chemical castration (group IV) enhanced colonic tumorigenesis, as indicated by increased prevalence, mean frequency and multiplicity of tumours. By contrast, surgical castration (group II) produced no significant change in tumorigenesis. Testosterone substitution (group III) reduced the prevalence of colonic tumours and resulted in a shift in tumour distribution into the left colon. The reduced tumour prevalence, however, appeared to be due to hormone vehicle, rather than testosterone, because group V also showed reduced prevalence and mean frequency of tumours.

*Invasiveness of colonic tumours >2 mm*

Castrated animals of group II, IV and V showed no significant differences from control group I. Surgical castration with hormone substitution led to a decreased proportion of invasive tumours in group III, which was significantly different from control animals ($P<0.05$) (Table II).

*Typing of invasive colonic tumours (tumour size >2 mm)*

A typical example of a tumour classified as adenocarcinoma is showed in Figure 1. Figure 2 depicts a mixed carcinoma. After chemical castration (group IV) an increased proportion of invasive tumours were classified as adenocarcinoma and signet ring cell carcinoma compared to control group I and all other groups. Hormone substitution after surgical castration (group III) increased the proportion of adenocarcinomas as compared to surgical castration alone (group II) (Table III).

*Grading of adenocarcinoma*

Colonic adenocarcinoma were graded as well and poorly differentiated tumours. The distribution of these two grades is shown in Table IV. In general, poorly differentiated tumours represented the majority of colonic adenocarcinomas. Castration with testosterone substitution resulted in increased percentage of poorly differentiated tumours ($P<0.02$).

**Table II** Invasiveness of colonic tumours (>2 mm)

| Group | Total number of tumours >2 mm (n) | Percentage of invasive tumours >2 mm (%) |
|---|---|---|
| I | 94 | 83 |
| II | 128 | 73 |
| III | 45 | 44[a] |
| IV | 261 | 81 |
| V | 55 | 63 |

$\chi^2$ test with Yates' correction: [a]$P<0.05$ *vs* groups I, II, IV.

**Table I** Number, prevalence, frequency, multiplicity site and distribution of colonic tumours (composed of data from Izbicki *et al.*, 1986)

| Group | Total number of tumours (n) | Prevalence of tumours (%) | Mean frequency of tumours (±s.d.) | Multiplicity of tumours (±s.d.) | Distribution of tumours: Right colon (%) | Left colon (%) |
|---|---|---|---|---|---|---|
| I | 118 | 87 | 3.1 ± 2.6 | 3.6 ± 2.5 | 52 | 48 |
| II | 145 | 93 | 3.6 ± 3.0 | 3.9 ± 3.0 | 50 | 50 |
| III | 50 | 71[d,f] | 2.1 ± 2.0 | 2.9 ± 1.8 | 26[b] | 74[b] |
| IV | 280 | 100[a,g] | 7.5 ± 4.5[c] | 7.5 ± 4.5[c] | 38 | 62 |
| V | 69 | 77 | 1.8 ± 1.6[e] | 2.3 ± 1.5[e] | 49 | 51 |

$\chi^2$ test with Yates' correction: [a]$P = 0.054$ *vs* group I; [b]$P<0.05$ *vs* groups I, II, V; [c]$P<0.05$ *vs* all groups; [d]$P = 0.053$ *vs* group II; [e]$P<0.05$ *vs* group IV; [f]$P<0.05$ *vs* group IV; [g]$P<0.05$ *vs* group V and III. Right colon defined as caecum, ascending colon, and transverse colon; left colon defined as descending colon and rectum.

**Figure 1** Histopathological section of a poorly differentiated adenocarcinoma ( × 180; haematoxylin and eosin).

**Figure 2** Histopathological section of a mixed carcinoma composed of areas of signet ring cell carcinoma and adenocarcinoma elements ( × 190; haematoxylin and eosin).

**Table III** Distribution of adenocarcinoma, mixed carcinoma and signet ring cell carcinoma

| Group | *Total number of invasive tumours (>2 mm) (n)* | *Adeno-carcinoma* | | *Mixed carcinoma* | | *Signet ring cell carcinoma* | |
|---|---|---|---|---|---|---|---|
| | | *n* | *%* | *n* | *%* | *n* | *%* |
| I | 78 | 58 | 74 | 20 | 26 | 0 | 0 |
| II | 93 | 62 | 67 | 28 | 30 | 3 | 3 |
| III | 20 | 16 | 80[b] | 4 | 20 | 0 | 0 |
| IV | 212 | 195 | 92[a] | 2 | 1 | 15 | 7[a] |
| V | 35 | 25 | 71 | 10 | 29 | 0 | 0 |

$\chi^2$ test with Yates' correction: [a]$P<0.05$ *vs* all groups; [b]$P<0.05$ *vs* group II.

*Epithelial proliferation in grossly normal mucosa*

*Crypt length* Hormonal manipulations resulted in significantly decreased crypt lengths in the right colon as compared to control group I. This effect was especially pronounced in chemically castrated animals (group IV). Hormone-substitution, however, did not result in an increase of crypt length. In contrast to the right colon, crypt length in the left colon did not exhibit significant changes after any type of hormonal manipulation (Table V).

*Number of cells per crypt* The mean number of cells per crypt in the right colon decreased as a consequence of hormonal manipulations only in group IV (chemical castration). In the left colon anti-androgen treatment (group IV) led to increased mean number of cells per crypt, thus indicating hyperplasia (Table VI).

**Table IV** Grading of adenocarcinomas

| Group | *Total number of adenocarcinomas (n)* | *Total number of well differentiated adenocarcinoma* | | *Total number of poorly differentiated adenocarcinoma* | |
|---|---|---|---|---|---|
| | | *n* | *%* | *n* | *%* |
| I | 58 | 20 | 35 | 38 | 65 |
| II | 62 | 20 | 32 | 42 | 68 |
| III | 16 | 0 | 0 | 16 | 100[a] |
| IV | 195 | 70 | 36 | 125 | 64 |
| V | 25 | 5 | 20 | 20 | 80 |

$\chi^2$ test with Yates' correction: [a]$P<0.02$ *vs* group I.

*Mitotic index*

*Right colon* In the right colon, chemical castration (group IV) led to a markedly increased mitotic index and shift of epithelial proliferation to include the upper third of the crypts. Other hormonal manipulations did not lead to significant changes of mitotic indices (Table VII).

*Left colon* Hormonal manipulations did not lead to significant changes of mitotic index as compared to control group I. Mitotic figures were present in the upper crypt third only in group I. Thus, only the right colon exhibited a shift of the proliferative zone to the upper crypt third after chemical castration, whereas in the left colon mitotic cells shifted to the middle third after chemical castration. However, after chemical castration (group IV) mitotic cells were predominantly located in the middle crypt third, as compared to control group I ($P<0.05$) (Table VIII).

**Table V** Crypt length in the right and left colon

| Group | *Right colon crypt length ± s.d. (μm)* | *Left colon crypt length ± s.d. (μm)* |
|---|---|---|
| I | 221 ± 51[a] | 239 ± 40 |
| II | 193 ± 43 | 237 ± 49 |
| III | 190 ± 45 | 248 ± 47 |
| IV | 179 ± 42[b] | 245 ± 51 |
| V | 192 ± 55 | 243 ± 39 |

One factorial analysis of variance: [a]$P<0.05$ *vs* all groups; [b]$P<0.05$ *vs* group I, II and V.

**Table VI** Number of cells per crypt in macroscopically normal mucosa

| Group | *Right colon mean no. of cells per crypt ± s.d.* | *Left colon mean no. of cells per crypt ± s.d.* |
|---|---|---|
| I | 36 ± 2 | 40 ± 5 |
| II | 35 ± 7 | 41 ± 6 |
| III | 35 ± 8 | 40 ± 5 |
| IV | 33 ± 8[a] | 44 ± 9[a] |
| V | 36 ± 9 | 41 ± 5 |

Kruskal–Wallis analysis and Mann–Whitney U test: [a]$P<0.05$ *vs* all groups.

**Table VII** Mitotic index and distribution of mitotic figures in the right colon of carcinogen treated rats

| Group | Mitotic index (mean ± s.d.) | Percentage distribution of mitotic figures in the crypt thirds | | |
|---|---|---|---|---|
| | | Lower | Middle | Upper |
| I | 12.1 ± 1.4 | 0[c] | 100 | 0[d] |
| II | 24.0 ± 1.9 | 8.7[c] | 82.6 | 8.7 |
| III | 9.2 ± 1.0 | 0[c] | 100 | 0 |
| IV | 38.4 ± 2.5[a] | 2.6[c] | 65.8 | 31.6[c,d] |
| V | 25.1 ± 1.8[b] | 24 | 64.0 | 12.0[c] |

Kruskal–Wallis analysis and Mann–Whitney U test: [a]$P<0.05$ *vs* groups I and III; [b]$P<0.05$ *vs* group III; [c]$P<0.05$ *vs* corresponding crypt third in the left colon of experimental group; [d]$P<0.05$ *vs* all groups.

**Table VIII** Mitotic index and distribution of mitotic figures in the left colon of carcinogen treated rats

| Group | Mitotic index (%) (mean ± s.d.) | Percentage distribution of mitotic figures in the crypt thirds | | |
|---|---|---|---|---|
| | | Lower | Middle | Upper |
| I | 23.3 ± 2.0 | 39.1 | 43.5 | 17.4 |
| II | 15.0 ± 0.9 | 40.0 | 6.0 | 0.0 |
| III | 25.2 ± 1.9 | 32.0 | 68.0 | 0.0 |
| IV | 23.4 ± 1.1 | 17.4 | 82.6[a] | 0.0 |
| V | 17.1 ± 1.6 | 41.2 | 58.8 | 0.0 |

Kruskal–Wallis analysis and Mann–Whitney U test: [a]$P<0.05$ *vs* groups I, II and V.

*Prevalence of dysplastic crypts*

Figure 3 shows a histopathological section with dysplastic crypts of the right colon of a carcinogen-treated rat. Generally, the prevalence of dysplastic crypts in the left colon tended to be higher than in the right colon; this was statistically significant for groups II and IV ($P<0.05$). Castration resulted in decreased prevalence of dysplastic crypts in the right colon as compared with control animals ($P<0.05$). Chemical castration, however, resulted in increased prevalence of dysplastic foci in the left colon of group IV ($P<0.01$ versus control) (Table IX).

**Figure 3** Histopathological section showing dysplastic crypts in the ascending colon of a carcinogen-treated rat (× 172; haematoxylin and eosin).

**Table IX** Prevalence of dysplastic crypts

| Group | No. of surviving animals (n) | No. of animals with dysplastic crypts in the right colon | | No. of animals with dysplastic crypts in the left colon | |
|---|---|---|---|---|---|
| | | n | % | n | % |
| I | 38 | 9 | 23.7 | 16 | 42.1 |
| II[a] | 40 | 2 | 5.0[b] | 14 | 35.0 |
| III | 24 | 10 | 41.7 | 12 | 50.0 |
| IV[a] | 38 | 14 | 36.8 | 30 | 78.9[c] |
| V | 39 | 9 | 23.1 | 19 | 48.7 |

$\chi^2$ test with Yates' correction: [a]$P<0.05$ for comparison of prevalence of dysplastic crypts in the right colon *vs* left colon of animals of group II and IV; [b]$P<0.05$ *vs* group I; [c]$P<0.01$ *vs* group I.

### Discussion

Human colonic carcinogenesis is thought to be influenced by androgens (Jacobson, 1984; Mehta *et al.*, 1980). A chemical insult to colonic mucosal cells is postulated to result in synthesis of specific androgen receptor proteins, rendering the mucosa cells hypersensitive to androgens (Jacobson, 1984). This hypothesis is supported by the detection of specific androgen receptor proteins in human colonic tumour tissue (Alford *et al.*, 1979).

Chemical induction of colonic carcinomas in rats has been found to provide a reliable model of colonic carcinogenesis in humans. Although an adenoma–carcinoma sequence cannot be identified as clearly as in humans (Maskens & Dujardin-Loits, 1981; Izbicki *et al.*, 1985), proliferative abnormalities similar to those observed in human individuals at high risk for colonic cancer are demonstrable (Izbicki *et al.*, 1988; Deschner, 1983). In recent years these experimental carcinogenesis models were used to evaluate the effects of steroid hormones, including androgens, and of hormonal manipulations on chemically induced tumours. Whereas some authors were able to demonstrate promoting effects of androgens (Moon & Fricks, 1977; Izbicki *et al.*, 1983; Mehta *et al.*, 1978), other authors presented conflicting results with protective effects of androgens against experimental colon carcinogenesis (Izbicki *et al.*, 1986).

Androgen receptors have also been studied in these experimental models. Mehta *et al.* (1980) found a carcinogen-induced induction of androgen receptors and postulated a maximum effect of androgens during the early phase of carcinogenesis. Whereas colonic mucosa of control animals which received no carcinogen treatment did not exhibit any specific androgen binding properties, carcinogen treatment was shown to result in the detection of specific androgen receptor proteins in macroscopically normal colonic mucosa (Mehta *et al.*, 1980). In addition, hormonal manipulations also influenced the amount of specific androgen binding sites. Gonadectomy led to detection of androgen receptors in colonic mucosa of control animals, suggesting an 'up-regulation' (Mehta *et al.*, 1980). Gonadectomised carcinogen-treated animals exhibited an even greater increase in receptor density in macroscopically normal mucosa as compared to gonadectomised control animals.

The highest concentration of specific androgen receptor proteins was detected in chemically induced colonic tumours (Mehta *et al.*, 1980). This increase of specific androgen-binding sites in chemically induced colonic tumours was also confirmed by Krelenbaum *et al.* (1984), Jacobson (1984) and Tutton & Barkla (1988).

Our group was unable to demonstrate a carcinogen-induced increase in androgen receptor binding sites in macroscopically normal colonic mucosa as compared to control animals. Colonic wall of control animals, which received the carcinogen vehicle only, exhibited androgen binding sites with typical characteristics of androgen receptors. Their density was not altered by carcinogen treatment (Izbicki *et al.*, 1986). These receptors were regulated, however, by the amount of circulating androgens. Receptor density in macroscopically normal colonic mucosa of carcinogen-treated animals was highly influenced by hormonal manipulations, confirming the 'up-regulation' which was postulated by Mehta *et al.* (1980). In contrast to previous findings (Mehta *et al.*, 1980; Krelenbaum *et al.*, 1984; Jacobson, 1984), we were unable to demonstrate an increase of specific androgen-binding sites in colonic tumours. Receptor density in tumours was generally 50% lower than in macroscopically normal colonic mucosa (Izbicki *et al.*, 1986). These conflicting results might be explained by different methods of receptor determination and different animal models (Izbicki *et al.*, 1986; Tutton & Barkla, 1988).

Because a receptor-mediated effect of circulating androgens on neoplastic events in the colonic crypt epithelium is postulated (Jacobson, 1984), the present study was designed to assess the effects of hormonal manipulations on cell proliferation, occurrence of dysplasia and tumour characteristics. In contrast to other authors (Moon & Fricks, 1977; Mehta *et al.*, 1978; Izbicki *et al.*, 1983), we did not find a promoting effect of androgens on experimental colonic carcinogenesis. Androgen administration in castrated rats resulted in decreased tumour prevalence. Concommitantly the proportion of invasive tumours was significantly lower in this group, thus suggesting a protective effect of androgens on experimental colonic carcinogenesis in this model. On the contrary, chemical castration with the androgenic progestin cyproterone acetate resulted in a significant increase in tumour prevalence, frequency and multiplicity, although a significant promoting effect on the proportion of invasive tumours was not observed. The hormonal manipulations produced only modest changes in the distribution of tumour types and grades. Interpretation of these conflicting results is difficult, as various strains of rats and chemical carcinogens were used in the different studies (Izbicki *et al.*, 1986).

In studies of the proliferation characteristics of chemically induced tumours, Tutton & Barkla (1982*a*, *b*) found significant effects of hormonal manipulations. Castration (chemical or surgical) of male and female animals resulted in a decreased rate of proliferation in tumours, whereas hormone substitution by testosterone propionate or oestradiol led to an increased rate of proliferation, also indicating a promoting effect of androgens.

In our study, hormone substitution in castrated rats significantly influenced the site distribution of colonic tumours, with tumours predominantly located in the left colon. This finding suggested different influences of androgens on proximal and distal colonic cancers (Izbicki *et al.*, 1986; Potter & McMichael, 1983). No significant effect on site distribution of colonic tumours was observed as a consequence of our other hormonal manipulations.

Proliferative abnormalities in colonic crypt epithelium are considered to be precursors to the malignant transformation of colonic mucosa in humans (Deschner, 1983) as well as in experimental animals (Deschner, 1983; Izbicki *et al.*, 1988). The postulated carcinogen-induced hypersensitivity of colonic mucosal cells to androgens (Jacobson, 1984) should therefore be reflected in alterations of proliferation as a consequence of hormonal manipulations.

In the ascending colon of carcinogen-treated control animals (group I) the overall proportion of mitotic cells was lower than in the descending colon ($P<0.05$). Thus the colonic segments showed a variable susceptability to the colonic carcinogen, as reported previously (Izbicki *et al.*, 1988). In the ascending colon of carcinogen-treated animals (group I), mitotic figures were detected only in the middle third of the crypt. By contrast, the descending colon exhibited the majority of mitotic figures in the lower and middle crypt thirds, with a smaller proportion in the upper crypt third. The proportion of mitotic cells in the lower and upper crypt thirds of the ascending colon was significantly different in comparison to the corresponding crypt thirds of the descending colon ($P<0.05$). The different distribution of mitotic cells in the colonic segments suggests a stage II proliferative abnormality in the ascending colon and stage III in the descending colon.

Castration of experimental animals by pharmacological means led to a significantly increased proliferation in the ascending colon, indicated by an increase in mitotic index to levels of over 15%. Hormone-substitution after castration resulted in decreased proliferation in the ascending colon to the level of carcinogen-treated control animals. Hormonal manipulations also influenced the distribution of mitotic cells in the epithelial crypts of the ascending colon. Chemical castration led to a significant shift of mitotic cells to the upper crypt third. A similar trend was observed after surgical castration although statistical analysis revealed no significant difference from control animals. On the contrary, hormone-substituted animals revealed a distribution of mitotic cells which was entirely similar to control animals.

By contrast with the right colon, hormonal manipulations did not dramatically alter the proliferative characteristics of the left colon as compared to control animals treated with the carcinogen only. No significant differences in mitotic index or distribution of mitotic cells were observed, except for a significantly higher proportion of mitotic cells in the middle crypt third after chemical castration.

The obvious different susceptability of the two colonic regions (Naito, 1982) is also indicated by the results of crypt length measurements. In the right colon, all hormonal manipulations led to significantly decreased crypt lengths, whereas the left colon did not reveal significant changes in crypt length after hormonal manipulations. Concommitantly with crypt shortening after castration, a significant decrease in epithelial cell numbers in the right colon of chemically castrated animals was evident. In the left colon, castration by surgical or pharmacological means was followed by hyperplasia of colonic crypts, as indicated by increased cell numbers and unchanged crypt length. Thus, we were unable to confirm the results of Tutton & Barkla (1982), who found significant alterations in crypt proliferation in the descending colon after hormonal manipulation, as assessed by estimation of mitotic rate after Vinblastine treatment. In contrast, our results clearly demonstrated significant proliferative alterations in macroscopically normal mucosa of the right colon only.

The prevalence of dysplastic crypts is generally thought to be influenced by the degree of proliferative abnormalities (Izbicki *et al.*, 1988; Deschner, 1983). In this study, severe proliferative abnormalities of the ascending colon, which were especially pronounced after chemical castration, were not followed by increased occurence of dysplastic crypts (groups II and IV). On the contrary, chemically castrated rats exhibited only slight changes in proliferative characteristics in the descending colon, but the highest prevalence of dysplastic crypts (group IV). A similar result was obtained after hormone substitution. Although hormonal manipulation did alter epithelial proliferation, these effects were not correlated to the occurrence of dysplastic crypts.

The site distribution of colonic tumours in right and left colon did not follow the site-specific prevalence of dysplastic crypts. Chemically castrated animals with a significantly higher prevalence of dysplastic crypts in the left colon exhibited the highest proportion of tumours in the left colon and the highest proportion of invasive tumours. Hormone-substituted animals showed an even distribution of dysplastic crypts, whereas colonic tumours were predominantly located in the left colon. Thus, prevalence of dysplastic crypts was not strongly related to tumour outcome in our study. The occurrence of site-specific effects of hormone manipulations on proliferative characteristics predominantly in the right colon, but not in the descending colon, further supports the hypothesis that cancers of the left and right colon may have different aetiologies, as was suggested by Potter and McMichael (1983).

The protective effects of the hormone vehicle on experimental colonic carcinogenesis remain unclear. Administration of the tenside Myri 53 induced changes similar to hormone substitution. Thus, a decreased prevalence, frequency and multiplicity of colonic tumours, as well as a lower porportion of invasive tumours, were found. A possible explanation could be a tenside-induced diarrhoea (Fitzhugh *et al.*, 1959), with consequent lower stool transit time as reflected by our own experience and the decrease of body weight (Izbicki *et al.*, 1986). This reduced transit time would result in a decreased faecal concentration of the carcinogen and a shortened contact time between colonic mucosa and carcinogen (Howe, 1982). Another explanation could be a lower food utilisation associated with intake of emulsifiers (Oser & Oser, 1956), as reflected by the decrease of body weight observed in our study. A reduced calorie intake is known to be associated with decreased colonic tumour incidence (Howe *et al.*, 1982).

In conclusion, the results of our study and those of other authors are at the present time too inconsistent to draw definite conclusions about the effects of hormonal manipulations. So far, our findings support the hypothesis of androgen sensitivity of colonic epithelium and of chemically induced colonic tumorigenesis in the rat.

This study was supported by the Deutsche Forschungsgemeinschaft grant no. Iz 1/1-1 and the Clayton Fund.

### References

ALFORD, T.C., DO, H.M., GEELHOLD, G., TSANGARIS, N.T. & LIPPMANN, M.E. (1979). Steroid hormone receptors in human colonic cancers. *Cancer*, **43**, 980.

AUTRUP, H. & WILLIAMS, G.M. (eds) (1983). *Experimental Colon Carcinogenesis*. CRC Press: Boca Raton.

BALISH, E., SHIH, C.N., CROFT, W.A. & 4 others (1977). Effects of age, sex and intestinal flora on the induction of colon tumours in rats. *J. Natl Cancer Inst.*, **58**, 1103.

DESCHNER, E.E. (1983). Adenomas: preneoplastic events, growth and development in man and experimental systems. *Pathol. Ann.*, **18**, 205.

FITZHUGH, O.G., BOURKE, A.R., NELSON, A.A. & FRAWLEY, J.P. (1959). Chronic oral toxicities of four stearic acid emulsifiers. *Toxicol. Appl. Pharmacol.*, **1**, 315.

HOLM, S. (1979). A simple sequential rejective multiple test procedure. *Scand. J. Stat.*, **6**, 65.

HOWE, G.R., MILLER, A.B., JAIN, M. & COOK, G. (1982). Dietary factors in relation to the etiology of colorectal cancer. *Cancer Detect. Prev.*, **5**, 331.

IZBICKI, J.R., DORNSCHNEIDER, G., HAMILTON, S.R., NAGELSCHMIDT, M. & WILKER, D. (1988). Sequential changes in colonic mucosal morphology and epithelial proliferation during chemically induced carcinogenesis in rats. *Dig. Surg.*, **5**, 99.

IZBICKI, J.R., HAMILTON, S.R., IZBICKI, W. & 4 others (1985). Lack of evidence for adenoma-carcinoma sequence in chemically induced colonic carcinogenesis in rats. *Dig. Surg.*, **2**, 143.

IZBICKI, J.R., SCHMITZ, R., KAMRAN, D. & IZBICKI, W. (1983). Androgens as promoters of colon carcinogenesis. *Cancer Detect. Prev.*, **6**, 355.

IZBICKI, J.R., WAMBACH, G., HAMILTON, S.R. & 4 others (1986). Androgen receptors in experimentally induced colon carcinogenesis. *J. Cancer Res. Clin. Oncol.*, **112**, 39.

JACOBSON, H.L. (1984). Present status of steroid hormone receptor in large bowel cancer. *Prog. Cancer Res. Ther.*, **29**, 367.

KRELENBAUM, M., KAREEM, A.M. FLEISZER, D. & FAZEKAS, A.G. (1984). Steroid hormone receptors in dimethylhydrazine-induced rat colonic tumours. *Anticancer Res.*, **4**, 395.

MASKENS, A.P. & DUJARDIN-LOITS, R.M. (1981). Experimental adenomas and carcinomas of the large intestine behave as distinct entities: most carcinomas arise *de novo* in flat mucosa. *Cancer*, **47**, 81.

MEHTA, R.G., FRICKS, C.M. & MOON, R.C. (1978). Role of dihydrotestosterone on 1,2-dimethylhydrazine-induced colon carcinogenesis. *J. Steroid Biochem.*, **9**, 856.

MEHTA, R.G., FRICKS, C.M. & MOON, R.C. (1980). Androgen receptors in chemically induced colon carcinogensis. *Cancer*, **45**, 1085.

MOON, R.C. & FRICKS, C.M. (1977). Influence of gonadal hormones and age on 1,2-dimethylhydrazine-induced carcinogenesis. *Cancer*, **40**, 2502.

NAITO, Y. (1982). Studies on experimental colon tumorigenesis in rats. 2. Cell kinetics of the colon epithelium and its relation to histogenesis of colon tumours. *Hiroshima J. Med. Sci.*, **31**, 51.

ODAGIRI, E., JIBIKI, K., DEMURA, R. & 4 others (1984). Steroid receptors and the distribution of IR-carcinoembryonic antigen in colonic cancer. *Dis. Colon Rectum*, **27**, 787.

OSER, B.L. & OSER, M.C. (1956). Nutritional studies on rats on diets containing high levels of partial emulsifiers – I. General plan and procedures; growth and food utilization. *J. Nutr.*, **60**, 367.

POTTER, J.D. & MCMICHAEL, A.J. (1983). Large bowel cancer in women in relation to reproductive and hormonal factors; a case–control study. *J. Natl Cancer Inst.*, **71**, 703.

TUTTON, P.J.M. & BARKLA, D.H. (1982*a*). The influence of androgens, antiandrogens and castration on cell proliferation in the jejunal and colonic crypt epithelium and dimethylhydrazine-induced adenocarcinoma of rat colon. *Virchows Arch. (Cell Pathol.)*, **3**, 351.

TUTTON, P.J.M. & BARKLA, D.H. (1982*b*). Differential effects of oestrogenic hormones on cell proliferation in the colonic crypt epithelium and in colonic carcinoma of rats. *Anticancer Res.*, **2**, 199.

TUTTON, P.J.M. & BARKLA, D.H. (1988). Steroid hormones as regulators of the proliferative activity of normal and neoplastic intestinal epithelial cells (review). *Anticancer Res.*, **8**, 451.